# Integrating telepathology and digital pathology with artificial intelligence: An inevitable future

**DOI:** 10.14202/vetworld.2024.1667-1671

**Published:** 2024-08-03

**Authors:** Alexandre Battazza, Felipe César da Silva Brasileiro, Ana Cristina Tasaka, Camilo Bulla, Pedro Pol Ximenes, Juliana Emi Hosomi, Patricia Fernanda da Silva, Larissa Freire da Silva, Fernanda Barthelson Carvalho de Moura, Noeme Sousa Rocha

**Affiliations:** 1 Department of Pathology, Faculty of Medicine of Botucatu, Sao Paulo State University (UNESP), Botucatu, Brazil; 2 Department of Veterinary Clinic, School of Veterinary Medicine and Animal Science, São Paulo State University, Botucatu, Brazil; 3 Department of Animal Science, Universidade Paulista, Sao Paulo, Brazil; 4 Universidade Municipal de São Caetano do Sul, Brazil; 5 Department of Pathobiology, College of Veterinary Medicine, Mississippi State University, Mississippi, USA

**Keywords:** artificial intelligence, diagnosis, telepathology

## Abstract

Telepathology and digital pathology, enhanced with artificial intelligence (AI), represent groundbreaking technology advancements. These entities offer information exchange, enhanced teaching and research, and automated diagnosis with high precision through a computerized approach. Machine learning in pathology shows promise for both human and veterinary medicine, yielding favorable results and in some cases, surpassing the accuracy of human pathologists. This study aimed to highlight the significance of integrated AI with telepathology and digital pathology, outlining both its advantages and limitations while emphasizing the crucial role of pathologists in its implementation. A literature review was conducted to uncover publications and data on telepathology and AI, and their implementation in human and veterinary medicine. This approach has facilitated information exchange, enhancing both teaching and research. In addition, it facilitates the creation of innovative methods and offers more precise patient diagnoses, adhering to ethical and legal standards. This study delivers valuable and comparable data on telepathology, digital pathology, and AI integration. Given the continually emerging nature of these technologies, further studies are essential for their application to human and veterinary medicine.

## Introduction

Telepathology, digital pathology, and artificial intelligence (AI) combine to revolutionize and reshape health systems through computerized approaches [1, 2]. According to Carlo Capè, global president and co-founder of Business Integration Partners, AI could eventually replace doctors in diagnosing health conditions due to its efficiency in performing tasks [3].

The combination of different tools with telepathology, such as AI, machine learning (ML), and deep learning (DL), can extract more layers of information from the whole slide image (WSI) and provide an image bank that can help future developers create an app that simplifies the interactions between pathologists, the diagnosis process, and the correlations between predictive markers and prognosis. This is only possible through artificial pixel pattern analysis, which is impossible with the human eye [4]. According to the Brazilian Industrial Development Agency (ABDI) survey, only 20% of young Brazilians know that future employment will be based on cutting-edge technologies [5], revealing a gap between young people’s knowledge and the demands of the productive sector. Telepathology is not as prevalent in emerging countries, such as Brazil and India. The limited percentage of government and business research investments in telepathology in these countries may be the primary reason for the limited development of scientific products and patents [6]. Education can help businesses adapt to new functions arising from digitalization and anticipate future threats to businesses easier through education [3]. In addition, with the emergence of free online tools for use in data banks, especially AI and bioinformatics, these tools can be continuously altered to provide democratic access to science [6].

This study explores the integration of AI, telepathology and digital pathology as a rising and unavoidable technology in both human and veterinary medicine. The review scrutinized peer-reviewed scientific studies in PubMed Central, published in Portuguese or English from 1998 to 2023. The text focuses on digital pathology, telepathology, AI, ML, and DL. Only publications in Portuguese or English were included. Online resources, including full-length articles and abstracts, were considered.

## Digital Pathology and Telepathology

Histological slides can be scanned and visualized digitally for consultation, collaboration, research, and education of students and residents [5, 7]. Digital histological sample processing encompasses image analysis, communication, and management [7]. These laboratories enhance the precision, consistency, and applicability of histological samples. Telepathology can be used to transmit this information to distant pathologists [8].

Telepathology has a long history dating back to 1968 when photos of black-and-white blood smears and urine samples were sent from Logan Airport in Boston to the General Hospital in Massachusetts for interpretation. The historical achievements are listed in Table-1 [7]. Telepathology involves four modalities: Static, dynamic, hybrid, and WSI [8].

**Table-1:** History of telepathology [7].

Year	History
1968	Sending black and white photos of blood smears through video from Logan Airport to Massachusetts General Hospital in Boston.
1980	Remote telepathology transmission demonstration on a commercial scale.
1986	First robotic video telepathology system using satellite. The first patent application for telepathology was prepared for filing at the US Patent and Trademark Office, which was granted in 1993.
1989	Norway’s national telepathology program for freezing-cutting services was established
1990	Published telepathological experience with over 2200 hospital cases from the US Department of Veterans Affairs.
1994	Availability of hardware for a complete telepathology system.
1995	Start of the Federal Administration of Public Income (AFIP) static image query service.
2000	The WSI system hits the market.
2001	Use of dynamic telepathology in the US Army Telemedicine Program.
2005	Conversion of the US Army to WSI platform.
2009	Approach to the use of digital pathology for primary diagnosis at the Food and Drug Administration (FDA) panel meeting.
2011	Introduction of WSI Dynamic-Robotic/Static Imaging Systems.
2013	Development of telepathology guidelines by the Royal College of Pathologists.
2014	Updating the Air Transport Association (ATA ’s) clinical guidelines for telepathology. Publication of the Canadian Association of Pathologists’ guidelines for establishing a telepathology service in anatomical pathology using WSI.

WSI=Whole slide imaging

## AI and ML

Understanding the association between digital pathology and AI can help explain certain concepts. AI is defined as the capacity of a machine to execute cognitive tasks to achieve a predetermined goal based on the provided data. This is possible because of the construction and programming of computers and learning robots [9].

ML techniques can be categorized according to the degree of human intervention. The manual method entails extracting an infinite number of characteristics explicitly defined in the dataset, drawn from experts’ qualitative thinking and reasoning during diagnosis [8].

Automatic techniques involve raw data processing as a part of the learning process, where the algorithm “learns” and later adapts to extract the characteristics without previous knowledge of the data. DL is an example of an automatic technique that is frequently used in convolutional neural networks and is efficient for applying scanned images and pattern recognition. This improves performance as long as more data are available [8].

## ML and DL Research

Previous research by Olsen *et al*. [10] has documented DL’s findings. Olsen *et al*. [10] achieved outstanding accuracy in the classification of nodular basal cell carcinoma, dermal nerves, and seborrheic keratosis in WSI slides using DL, with rates of 99.45%, 99.4%, and 100%, respectively. Recently, Erdem and Bozkurt [5] achieved an accuracy of 97% and an area under curve (AUC) of the receiver operating characteristic curve of 0.958 for classifying the same tumor type by comparing different approaches.

Kapatia *et al*. [11] developed an artificial neural network model capable of distinguishing pleomorphic adenomas from salivary gland adenoid cystic carcinomas, achieving an AUC of 1. Seven DL algorithms were assessed for diagnosing breast tumor metastasis in lymph nodes. It was noted that they surpassed 11 pathologists in diagnosis simulation with a time restriction, with the AUCs of the best algorithm and the best pathologists being 0.99 and 0.88, respectively [3].

The scope of ML research in veterinary pathology is significantly less than that of DL. In a feline intestinal disease study, ML algorithms were employed to enhance standardized histopathological reports by identifying credible microscopic characteristics [1]. The algorithm created by Hattel *et al*. [12] for categorizing micrographs of bovine liver, lung, spleen, and kidney tissues differentiates between normal, acute (acute, suppurative, and chronic), and chronic tissue necrosis/inflammation types.

## Use Perspective

The potential applications and relationship of these emerging technologies to pathology and digital pathology warrant examination. Telepathology’s benefits and drawbacks were succinctly presented by Farahani and Pantanowitz [7]. According to the World Health Organization [13], high cost, underdeveloped infrastructure, and insufficient knowledge and technical support hinder telepathology implementation in developing countries. In developed nations, legal issues surrounding patient confidentiality are a major concern [13, 14]. The United States, Germany, Italy, England, Canada, and France are heavily invested in telepathology. Developing countries such as Brazil and India hold significant roles [6].

Proper medical information transmission, image standardization, and minimizing sampling errors highlight the importance of organizational support in telepathology [15].

**Figure-1:**
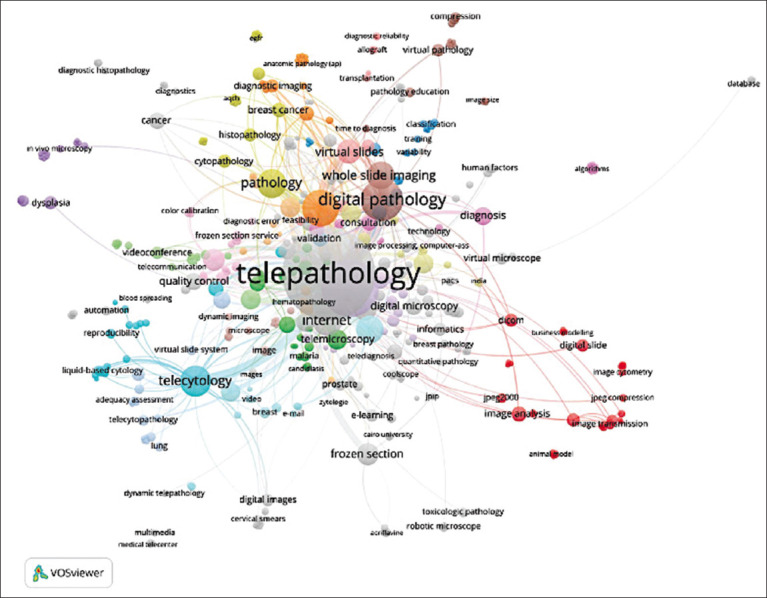
Use of telepathology in medicine [6].

The use of ML and DL to compare medical and non-medical data are a promising strategy for early cancer diagnosis [16]. Other applications already cited in human medicine include telepathology for telecytology, adequacy assessment, liquid-based cytology, hematopathology, diagnostic error feasibility, frozen sections, and virtual slides (Figure-1) [6]. However, it is necessary to access a large quantity of patient data and images to train AI algorithms [16].

Consequently, the small number of scanned images and lack of detailed and consistent data for ML training represent challenges in their broad usage. Thus, AI development must begin when substantial computational resources are available, and large datasets must be carefully identified [8].

Theoretically, an omnipotent AI that can replace pathologists is feasible; however, its implementation remains quite distant. Pathologists contribute their experience and pathophysiological knowledge, starting in the pre-analytical phase with sample quality assessment, followed by interpretation of the results and training using reliable information [7].

The integration of digital pathology with AI and the use of telepathology has expanded in recent years. This expansion enabled the exchange of information and improvements in teaching and research. Furthermore, it aids in new approaches and provides more detailed and accurate diagnoses of patients, obeying ethical and legal principles [7]. For example, whole-slide imaging is used as a tool for teaching students, long-distance networking, and business in veterinary medicine [17]. The UN Framework Convention on Climate Change COP 30 insights highlight the importance of sustainable development, and telepathology facilitates goals achieved through pollutant reduction in slide production and material sharing [18]. Some studies have used ML to support the conservation of wildlife species and environmental protection [17].

When a multihead microscope was introduced in the technology scenario of the pathology laboratory, the training and qualification process of postgraduate students and future pathologists was simplified and performed with more accuracy but was limited to those locations. WSIs allow the use and exploration of the same slides in different locations, which facilitates teaching and learning processes [19]. This also promotes the feasibility of diagnosis by allowing access to other pathologists without physically sending the slides to different locations. However, pathologists must have a basic knowledge of digital pathology, telepathology, and ML systems and their advantages and limitations to fit into future scenarios in which diagnosis involves computer science [4].

However, pathologists must have a basic understanding of digital pathology, telepathology, and ML system’s advantages and limitations to fit into future scenarios in which diagnosis also involves computer science [7]. Granter *et al*. [16] analyzed the performance of an AI system called AlphaGo and predicted that AI could eventually replace even the most subtle histopathological analyses. In another study, it was estimated that electron microscopy would remain in use for approximately 150 years.

In 2020, Brazil regulated the use of telemedicine in human medicine through a resolution issued by the Federal Council of Medicine. In the same year, public representatives favored the use of telemedicine during the COVID-19 pandemic. The Federal Council of Veterinary Medicine recognizes the importance of discussing veterinary topics with veterinarians and has authorized the use of telemedicine by veterinarians since 2022 [2, 9, 20, 21]. Telepathology, when used concomitantly with ML and DL, can increase the observation of patterns that can facilitate more precise diagnosis and future research to correlate with clinical information and predict patient prognosis [19, 22]. Studies in veterinary medicine are scarce, and this area should be further explored to improve animal welfare and public health.

## Conclusion

ML’s ability to analyze image patterns surpasses that of the human eye, enabling precise image criteria for diagnosis. AI integration allows for consistent and relevant data covering telepathology and digital pathology to be provided. The review presents definitions and tools that aid in the integration of telepathology and digital pathology with AI. However, further research is necessary since these technologies are advancing and will eventually be applied in human and veterinary medicine.

## Author’s Contributions

AB, FCSB, and FBCM: Performed data acquisition and analysis; PPX, JEH, PFS, and LFS: Wrote the manuscript. ACT, CB, and NSR: Reviewed the manuscript. All authors have read, reviewed, and approved the final version of the manuscript.
